# Evaluation of genomic island predictors using a comparative genomics approach

**DOI:** 10.1186/1471-2105-9-329

**Published:** 2008-08-05

**Authors:** Morgan GI Langille, William WL Hsiao, Fiona SL Brinkman

**Affiliations:** 1 Department of Molecular Biology and Biochemistry, Simon Fraser University, Burnaby, BC, Canada

## Abstract

**Background:**

Genomic islands (GIs) are clusters of genes in prokaryotic genomes of probable horizontal origin. GIs are disproportionately associated with microbial adaptations of medical or environmental interest. Recently, multiple programs for automated detection of GIs have been developed that utilize sequence composition characteristics, such as G+C ratio and dinucleotide bias. To robustly evaluate the accuracy of such methods, we propose that a dataset of GIs be constructed using criteria that are independent of sequence composition-based analysis approaches.

**Results:**

We developed a comparative genomics approach (IslandPick) that identifies both very probable islands and non-island regions. The approach involves 1) flexible, automated selection of comparative genomes for each query genome, using a distance function that picks appropriate genomes for identification of GIs, 2) identification of regions unique to the query genome, compared with the chosen genomes (positive dataset) and 3) identification of regions conserved across all genomes (negative dataset). Using our constructed datasets, we investigated the accuracy of several sequence composition-based GI prediction tools.

**Conclusion:**

Our results indicate that AlienHunter has the highest recall, but the lowest measured precision, while SIGI-HMM is the most precise method. SIGI-HMM and IslandPath/DIMOB have comparable overall highest accuracy. Our comparative genomics approach, IslandPick, was the most accurate, compared with a curated list of GIs, indicating that we have constructed suitable datasets. This represents the first evaluation, using diverse and, independent datasets that were not artificially constructed, of the accuracy of several sequence composition-based GI predictors. The caveats associated with this analysis and proposals for optimal island prediction are discussed.

## Background

Bacteria are the most abundant Domain of life that exists on earth (based on biomass) [1]. The species we see today are highly diverse, reflecting adaptations to a wide range of environments over billions of years. One of the major sources of adaptability for bacteria is the ability to obtain genes horizontally from other sources, including other prokaryotes, viruses, and even eukaryotes [2]. Analysis of bacterial genomic sequences has indicated that many of the horizontal gene transfer (HGT) events observed in bacteria involve clusters of genes. Collectively, these genomic regions are referred to as genomic islands (GIs) [3]. GIs, which range in size from ~5–500 kb, have become of significant interest, since they are frequently observed to encode genes involved in particular adaptive functions of medical and environmental importance [4,5]. The concept of GIs was derived from the term pathogenicity island, which was initially coined by Hacker and colleagues to describe a genomic region of uropathogenic *Escherichia coli *that harbours clusters of virulence factors that can be spontaneously deleted [6]. These regions also exhibit other sequence and annotation features that can be used to distinguish them from the rest of the genomes, as described below [3]. Subsequently, the term GI was adapted to describe genomic regions that contain similar features as PAIs but encode gene products of other functions. Reflecting the wide variety of genes found in GIs, function-specific terms such as "antibiotic resistance islands" (encode antibiotic resistance genes), "fitness islands" and "metabolic islands" (encode adaptive metabolic properties such as phenolic compound degradation) have been invented to describe different types of GIs [3].

Previous characterization of GIs has identified many sequence and annotation features that are frequently associated with GIs, such as differing sequence composition (%G+C, dinucleotide bias, codon usage bias, etc.), flanking direct repeats, and the presence of mobility and tRNA genes in the region [4,7-9]. Computational tools have therefore been developed to aid the identification of islands in genomic sequences that are based on sequence composition analysis [10-16]. These tools rely on the observation that different organisms exhibit different nucleotide pattern preferences that constitute their genome signatures. More closely related organisms are assumed to share similar preferences and, therefore, have more similar signatures. As a result, for a gene-cluster whose signature deviates from the genome signature, a plausible explanation is that this gene cluster has a foreign origin and its signature reflects that of the original donor. The tools, therefore, in general calculate the frequencies of oligo-nucleotides (typically ranging from 2 to 9) for a sub-region of a genome and compare these results with the expected frequencies from that genome. One advantage of this approach is that GI prediction can be made from a single genome sequence. However, GI predictors based on sequence composition may lead to false positive GI predictions due to other factors causing a bias in sequence composition, such as high gene expression level [17]. Furthermore, these approaches may miss the identification of GIs that have been acquired from genomes with similar sequence composition or more ancient GI acquisition events that may have ameliorated to the host genome sequence composition over time [18].

An alternative approach that is independent from sequence composition-based approaches is to use comparative genomics to identify genomic regions that have a clear phyletic pattern of non-vertical inheritance. In these methods, putative GIs are often defined as clusters of genes in one genome that are not present in a related genome. They are based on the observation that GIs are sporadically distributed among closely related species or strains and can sometimes be found between very distantly related species as judged by the degrees of sequence divergence in 16S rRNAs or other orthologs [19]. Until recently, this approach could only be performed manually for a few species that had enough similar sequenced genomes [20-25]. However, recent research has started to utilize the increasing number of completely sequenced genomes by constructing tools that can perform large-scale genomic comparisons, such as MobilomeFINDER [26] and MOSAIC [27]. MobilomeFINDER uses whole genome alignments to identify GIs, but is similar to Islander [13], such that it is limited to identifying only islands that have inserted in disrupted tRNAs. Although MOSAIC identifies strain specific regions that have not necessarily inserted into a tRNA, it can mistakenly identify inversions and translocations between genomes as strain-specific regions. In addition to these limitations, all current comparative genomics based GI prediction tools require the manual selection of both the query and the comparative genomes as input, which may result in inconsistent selection criteria due to the unfamiliarity of different phylogenetic distances within genera.

We now report here the development of "IslandPick", the first completely automated comparative genomics approach to identify GIs. Starting with all sequenced bacterial genomes as input, we use stringent but potentially flexible criteria, with distance cutoffs, to select query genomes that have a sufficient number of suitably related species or strains to conduct an analysis of GIs. We used this IslandPick method to identify datasets of GIs from several genomes as well as a dataset of conserved backbone genomic regions that are probable non-GIs. We evaluated how well these datasets agreed with GIs reported in the literature. We also evaluated how well these datasets agreed with those predicted using previously published sequence composition-based GI tools since our comparative genomics based method is independent of sequence composition-based methods. Analyses of GI/HGT prediction tools have been previously published, but have used either artificial datasets or real data from only a few species [15,28]. Our IslandPick method reported here focuses on developing robust positive and negative datasets that can be used as an independent non-artificial benchmark for previous and future GI prediction tools. Moreover, as additional genome sequences become available, IslandPick can be applied to those genomes to expand the benchmark dataset in a consistent and automated fashion.

## Results

### Genomic island predictions

**Figure 1:**
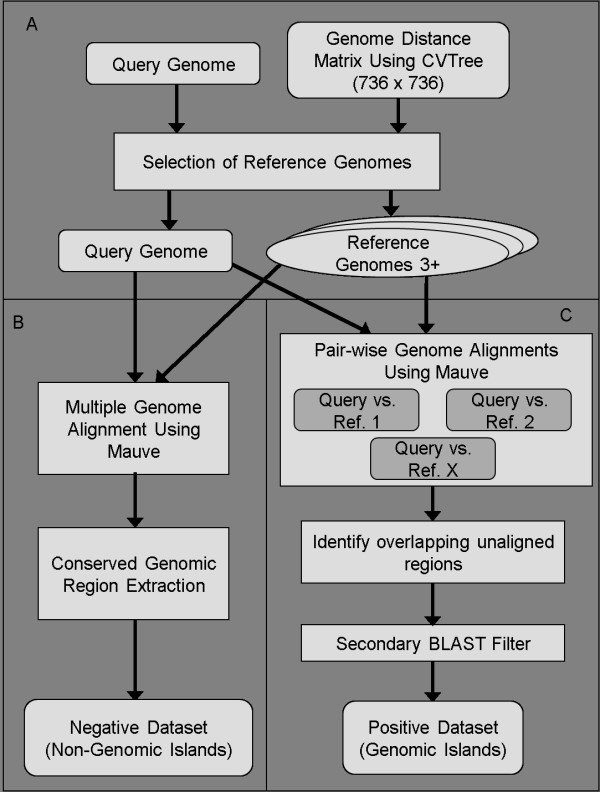
**Pipeline of how negative and positive datasets of GIs are derived given a single query genome as input**. A pre-computed genome distance matrix using CVTree is required as input as well as the query genome A). If there is enough suitable reference genomes selected for comparison with the query genome then the query genome and reference genomes are used in a Mauve multiple genome alignment and all conserved regions are extracted into a negative dataset of GIs B). The positive dataset is constructed by taking each query genome and aligning it pair-wise with each reference genome. Then all unaligned overlapping regions found in the query genome from the pair-wise alignments are filtered using the NCBI BLAST to ensure that they are truly unique to the query genome C).

In order to evaluate the sensitivity/recall of previously developed GI predictors based on sequence composition bias, we constructed a dataset of putative GIs based on a high-throughput comparative genomics approach that we developed. GIs were identified as regions that were present only in the genome being queried when compared to multiple genomes from closely-related species or strains (see Methods: Detecting GIs using comparative genomics). Of the 675 completely sequenced microbial genomes, 736 chromosomes were initially used as queries in our pipeline (Fig. 1). 377 of these did not have at least 3 related species/strains while many others did not meet our stringent criteria to do a comparative analysis. However, as more genome sequences become available, the number of genomes matching our criteria is expected to improve. We found that 173 chromosomes met our requirements for the prediction of GIs and conserved backbone sequences. A subset of 134 chromosomes contained GIs while our method did not detect GIs using our method in the other 39 chromosomes [see Additional file 1]. Many of these 39 genomes may contain GIs that are smaller than 8 kb or have other cases of HGT that were not being targeted by our approach. The dataset was further reduced to 118 chromosomes, because a negative dataset could not be predicted for 14 chromosomes and the GI prediction tool SIGI-HMM gave errors on another 2 chromosomes (see below). In total, we identified 771 GIs, comprising 12.4 Mb and ranging in size from 8–31 kb, within 118 chromosomes from 117 different strains and 22 genera [see Additional file 2]. These putative GIs contained a total of 11,404 annotated genes with an average of 14.8 genes/GI and 97.5 genes/strain [see Additional file 3].

### Negative Dataset

In order to evaluate the specificity/precision of previously developed GI predictors we constructed a dataset of genomic regions that were not likely to contain GIs. We adapted our GI prediction pipeline to identify large genomic regions that were conserved in several genomes (see Methods: Detecting highly conserved regions (non-GIs)). These conserved genomic regions were identified for the same 134 query genomes that we used for prediction of GIs. We could not identify any conserved regions larger than 8000 base pairs for 14 of these chromosomes, using our conservative criteria, and so these were removed from both the positive and negative datasets. The resulting negative dataset was about 4 times larger than our dataset of GIs, containing approximately 50.6 Mb over 3770 separate genomic regions [see Additional file 4]. The size difference between the negative and positive datasets was expected since the proportion of HGT versus conserved backbone in a genome is normally much smaller [29-31].

### Comparison with previous literature

Although, there is no gold standard dataset of GIs, we wanted to examine how previously published GIs overlapped with our datasets. We identified 5 strains from our list of 117 that had published GIs [21-25]. As with the analysis of the sequence composition based GI predictors (see Results: Comparison to sequence based approaches), we calculated the overlap of the published GIs against our positive and negative dataset. We found, potentially due in part to the similar manual comparative genomics methods sometimes used to identify GIs in the literature dataset, that the literature GIs had the most agreement with our datasets (versus the GI predictors evaluated below). Literature GIs had the highest precision, recall, and overall accuracy of 100, 87%, and 96%, respectively, when using IslandPick-predicted islands as the text dataset (Table 1).

**Table 1:** Average number of GI predictions and accuracy measurements of several GI prediction tools.

**Tool**	**Average number of nucleotides in GIs per genome (kb)**	**Precision**	**Recall**	**Overall Accuracy**
**SIGI-HMM**	232.7	92.3	33.0	86.3
**IslandPath/DIMOB**	170.7	85.8	35.6	86.2
**PAI IDA**	163.2	68.0	32.2	83.7
**Centroid**	171.3	61.3	27.6	82.4
**IslandPath/DINUC**	444.4	54.8	53.3	82.2
**Alien Hunter**	1264.8	38.0	77.0	70.8

**Literature**	639.4	100	87.0	96.3

*Results are averaged from 118 chromosomes in 117 different strains [see Additional file 6] except for the "Literature" GIs, which were averaged over 5 strains; *Escherichia coli *O157:H7, *E. coli *O157:H7 EDL933, *Salmonella enterica *Typhi str. CT18, *S. enterica typhimurium *LT2, and *Streptococcus pyogenes *MGAS315 [21-25].

### Comparison to sequence composition based approaches

The GIs and the non-GIs identified from our comparative genomics approach were used as positive and negative datasets, respectively, to evaluate the accuracy of several previously published sequence based GI prediction tools [8,12,15,16,29]. The tools were run using their default parameters on the same 118 chromosomes and any overlapping regions with the negative dataset were considered false positives while overlapping regions with the positive dataset were considered true positives [see Additional file 5]. The following accuracy calculations were measured using the number of overlapping nucleotides [see Additional file 6]; although results were not significantly different when counting only GIs with greater than 50% overlap (data not shown). We found that the precision and recall for the tools evaluated varied considerably (Table 1). SIGI-HMM performed the best with 92% precision (though only 33% recall) whereas AlienHunter had the best recall at 77% (though only 38% precision). SIGI-HMM and the IslandPath/DIMOB tool had comparable overall highest accuracy of 86% with IslandPath-DIMOB more suitable for analyses requiring a slightly higher recall (precision of 86% with a recall of 36%). All of the tools had similar overall accuracies ranging from 82–86% (but with differing emphasis on precision versus recall) except for AlienHunter, which had an accuracy of only 71%. This appeared to be primarily due to the large number of predictions being made by AlienHunter (1264.8 kb of GI/genome) versus the other methods (163.2 to 444.2 kb of GI/genome).

For completeness, we also calculated the accuracy of each tool using every other tool as the benchmark [see Additional file 7]. The average accuracy measurements over all benchmarks for each tool were very similar to those calculated using only our datasets indicating that the datasets generated using our comparative genomic approach may be an appropriate reference dataset for future use. These positive and negative GI datasets, and the source code for development of these datasets, are available at .

### Comparison of sequence composition based approaches using additional GI datasets constructed with more relaxed criteria

IslandPick defaults can be modified to allow the prediction of GIs with more ancient origins, and so we created two additional datasets based on the selection of more divergent genomes for use in GI and non-GI prediction (see Methods). The first "relaxed" dataset had approximately 46% more GIs predicted per genome, while as expected the negative datasets stayed about the same size with a 3% increase in the relaxed dataset. Notably, accuracy relative to the literature dataset went down slightly [see Additional file 8 and Additional file 9], indicating that our IslandPick defaults do most accurately reflect literature-based GI data. The sequence composition-based tools also all had a relative decrease in accuracy using this more relaxed dataset: Accuracy decreased between 4.5 and 6.6% for all methods, with the exception of Alien Hunter (the method with highest recall but lowest precision) which showed the smallest decrease of 0.6% [see Additional file 8 and Additional file 9]. Using a second more relaxed dataset of parameters resulted in yet another decrease in predicted accuracy of the GI tools and the accuracy relative to the literature-based dataset also decreased further (data not shown). While the use of more relaxed criteria for GI prediction may still have its uses, our results indicate that the default settings of our IslandPick method are most appropriate for predicting islands that most closely resemble what is reported in the literature. Also, the sequence composition-based methods appear to perform best when using the default IslandPick-predicted GI datasets for evaluation.

## Discussion

We have introduced and outlined, IslandPick, a novel automated method for predicting GIs using comparative genomics. We have used IslandPick, with its stringent default criteria, to generate test datasets of GIs and non-GI regions that are used to evaluate the accuracy of multiple sequence composition based GI predictors. This represents the first evaluation of GI predictors based on real (non-artificial) GI data from several different strains of bacteria [15,28]. For organisms that have suitable sequenced genomes for comparison, we identified very probable GIs and genomic regions that were very probable non-GIs. By developing separate negative and positive datasets that were independent of sequence composition based approaches we were able to assess the accuracy of several GI predictors.

According to our analysis, SIGI-HMM has the highest precision and shares comparable overall accuracy with IslandPath/DIMOB which has higher recall at the expense of precision. SIGI-HMM is the only tool tested that measures codon usage and notably it also identifies codon usage associated with highly expressed genes and then discards such genes from the analysis. While more study is needed, this suggests that regions displaying codon usage bias of a pattern that is not associated with highly expressed genes are more likely to be GIs. Consistent with this, the IslandPath/DIMOB method that requires both a dinucleotide bias and the presence of a mobility gene for a GI prediction does much better than the IslandPath/DINUC method which measures only dinucleotide bias. The latter can result in false positives from highly expressed genes but higher predictive recall/sensitivity. AlienHunter had the lowest precision (38%); however, it had by far the highest recall value (77%) with more than twice as many predictions as any other tool.

Based on these results we suggest the use of SIGI-HMM for making very precise predictions where a high confidence dataset of GIs is preferred while AlienHunter can be used as a first-pass tool to capture most GIs for further refinement. If suitable comparative genomes are available, IslandPick would be a top choice for GI prediction, however it should be emphasized that IslandPick is at this time really designed for generating robust island datasets for evaluating GI predictors, rather than being a GI predictor itself. Its utility as a GI predictor could increase though as more genome sequences, suitable for a comparative genomics approach, are made available. If comparative genomes are not available, the results generally suggest that by combining multiple features of GIs, as in the IslandPath/DIMOB dataset, and accounting for highly expressed genes, which SIGI-HMM does and IslandPath/DIMOB does indirectly, a better overall predictor could be created. More analysis of the differences in sequence composition between true positives and false positives in this analysis could be insightful.

Our results show that all GI predictors had a decrease in overall accuracy when trying to predict more ancient islands. Considering that sequence composition based predictors would have trouble detecting significant signals in older GIs due to amelioration to the host genome, it was not surprising that the overall accuracy for all tools decreased [18]. Alien Hunter had the lowest decrease in overall accuracy however it still maintained the lowest precision and overall accuracy for the prediction of this dataset and SIGI-HMM still out performed the other sequence composition-based tools for predicting these more divergent islands. It is possible that the accuracy of some of these sequence composition-based tools could be improved by optimizing their parameters. However, out of all the tools, SIGI-HMM and Centroid were the only ones with a clearly defined sensitivity/statistical parameter and even for these there were no recommend suggestions besides the default. Although default parameters for all tools are presumably maximized to result in the best overall accuracy, some fine tuning may improve their results.

Of course there are clear limitations to predicting GIs using comparative genomics in order to produce the GI dataset used in our evaluation. The choice of reference genomes for comparison to each query genome can result in differences in the positive and negative datasets. In our genome selection we use several distance cutoffs to minimize this bias as much as possible (example given in the next paragraph). GIs could be present in the negative dataset if a GI was inserted before the divergence of all genomes being examined. To minimize these in our datasets we require that at least 3 reference genomes be used for each query genome and that at least one reference genome is at least some minimal distance away from the query genome. Similarly, we minimize the number of false positives in our positive dataset by requiring that the putative GI is present only in the query genome compared to all reference genomes. Therefore, a deletion of the same genomic region would need to occur independently in 3 or more strains for it to be mis-predicted as a GI in our analysis. Similarly, a GI that inserted into multiple genomes would have to be conserved in all of the diverse genomes studied to be improperly placed in the negative dataset. Although using several rules in our genome selection process results in very stringent datasets of GI and non-GI regions, it does limit the number of organisms that can be used by IslandPick. Relaxing the genome selection process by the removal of some of these cut-offs would allow our approach to be applicable to more genomes. For example, by requiring only 3 genomes within a certain maximum distance we would increase the number of chromosomes usable by IslandPick from 173 to 359, based on the currently available genome sequences. It should be emphasized that IslandPick was not developed to be a GI prediction tool that would replace sequence-based composition tools, which can be used on any genome without the requirement of having several other comparative genomes; rather, our IslandPick approach allows the testing of these tools and in certain cases can also be used for GI prediction (cases that should increase notably in the future, as more and more genomes are sequenced).

The absence of large islands (> 30 kb) in our positive dataset is probably due to the fact that only a few similar genes between genome regions would cause our method to split a large island into two smaller ones. Considering that as an island gets bigger there is a greater chance of detecting some similarity between the genomic regions being compared, we would expect that very large GIs may be split into smaller ones. As indicated in recent research, this limitation could be improved in the future by spanning together islands that are interrupted by only small regions of low similarity [28]. It must also be appreciated that the GI regions identified represent a set of GIs that were acquired within a particular window of divergence of the strains being examined. Any genomic regions that did not have clear evidence of GI or non-GI status were not included in either of the datasets so that tools that predicted such possible/uncertain GIs were not penalized. This would include GIs that have inserted into multiple strains or those that have partial similarity with other genomic regions. Rather, our methodology penalizes tools that falsely predict GIs in highly conserved backbone regions that very likely do not contain true GIs, and also the method penalizes tools that don't predict a subset of GIs that are very likely true positives. In fact, our approach produced the smallest dataset of GIs compared to all of the methods we used in this study [see Additional file 5] and the proportion of the genomes that are covered in both of our positive and negative datasets combined, ranges from 10%–30% per genome. Therefore, we do not make predictions for the majority (70%–90%) of the genome, reflecting the high accuracy of our positive and negative datasets using our cutoffs. Also, our comparative genomics-based GI datasets had the highest agreement with the smaller curated, literature-based dataset.

As an example of the IslandPick approach, when *Salmonella enterica *Typhi CT18 is used as a query genome to identify islands using our default cutoffs, very closely related genomes including *S. enterica *Typhi Ty2 and *S. enterica *Paratyphi A str. ATCC 9150 were excluded from comparison. Therefore, we identify GIs that have inserted after the divergence of *S. enterica *Typhi CT18 and the next most related genome that has been sequenced, which is *S. typhimurium *LT2. Islands that inserted before the divergence of CT18 and LT2 would also not be included in our positive dataset, using these stringent cutoffs. However, we require that at least one genome be a certain distance from the query genome (*Shigella dysenteriae *Sd197 in this example), so that these more ancient GIs are not improperly included in the negative dataset. We assume that any sequences shared between the query genome (e.g. *Salmonella enterica *Typhi CT18) and the comparative genomes including those that meet the single distant genome cutoff (e.g. *S. dysenteriae *Sd197) are sufficiently stable and can be considered as the conserved genome backbone. Again, distance cutoffs can be modified in IslandPick to detect more ancient islands or those acquired more recently.

Similar to other GI prediction tools we do not try to identify the origins or the methods of horizontal transfer for these GIs. Indeed future research on many of these large regions of HGT will likely allow them to be sub-classified into known mobile elements such as conjugate transposons, integrated plasmids, integrons, and prophage; and will depend on robust prediction tools and knowledge of their strengths and weaknesses. Also, new sequence composition based GI prediction tools will likely combine components from previous methods to maximize both precision and recall [9]. Comparative genomics studies like this one, will aid in these areas by providing an independent method for GI prediction. As more genomes are sequenced, the distance cutoffs used in this method should be re-evaluated, but this overall approach should only increase in utility as the number of completely sequenced microbial genomes increases into the thousands. This analysis of the accuracy of composition-based GI predictors should aid both development and use of such predictors, which are becoming of increasing importance as the critical role of GIs in microbial evolution becomes more apparent.

## Conclusion

We have demonstrated, through the comparison of our IslandPick method with a reference literature dataset of GIs, that the prediction of GIs can be performed using a fully automated comparative genomics approach. We produce reference GI datasets (positive and negative) using IslandPick that allow an independent analysis of the accuracy of several previously published sequence composition-based GI prediction tools. Our analyses of the accuracy of GI predictors should aid researchers in formulating an appropriate approach to identify GIs, based on whether they prefer high recall/sensitivity or precision/specificity. Such GI predictors are likely to become of increasing importance in bacterial genome analysis, as appreciation grows of their significant role in adaptations of medical and environmental importance.

## Methods

### Genome data source

All 675 complete bacterial genome sequences available at the time of this study were obtained from the National Center for Biotechnology Information (NCBI) FTP server [32] and stored locally as of May 4, 2008. These genome sequences comprised a total of 1225 replicons of which 736 were chromosomes. All 736 chromosomes were treated as independent units from each other in all analyses.

### Automated comparative genome picking

An overview of our approach is illustrated in Fig. 1. We used an external application called CVTree, which infers evolutionary relatedness based on oligo-peptide content of complete predicted proteomes, to establish relative-phylogenetic distances between organisms. The source code for CVTree [33] was obtained and used to calculate the 270,480 distances between every pair of bacterial chromosomes; requiring approximately 526 hours (or ~7 seconds per calculation) of computation time (based on a single Intel Xeon 2.8 GHz machine) or ~4 hours on a 130 node cluster. The distances outputted by CVTree are on a scale and range between 0 and 0.5. To ensure that CVTree was behaving suitably, we compared these distance calculations to those produced by more conventional phylogenetic distance measures using PHYLIP [34], using *carB *and *omp85 *genes as reference sequences, as we have used previously for phylogenetic analysis of species [35]. Other approaches were tested to calculate evolutionary distances, such as SHOT [36], however CVTree was found to give the most consistent results (data not shown).

**Figure 2:**
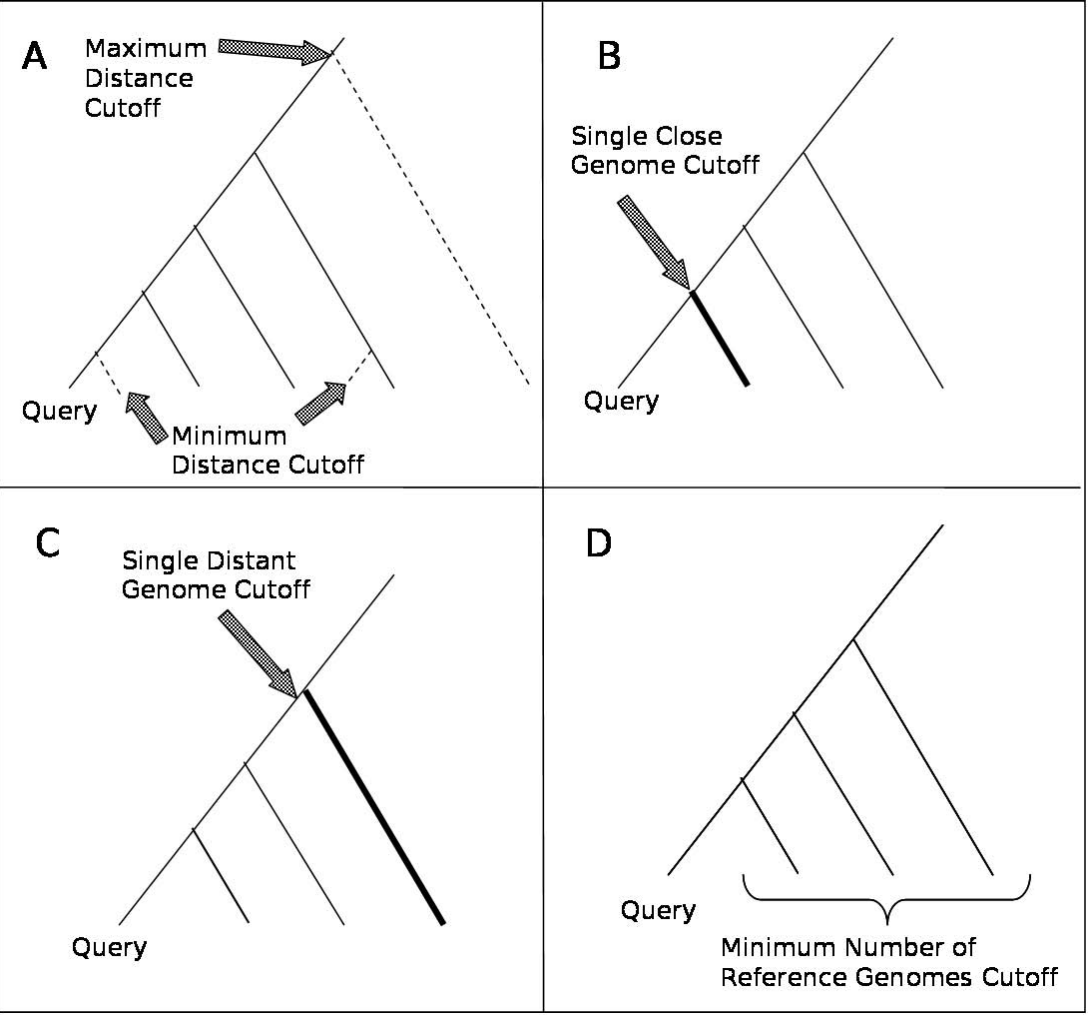
**Effect of cutoffs on a sample genome tree**. First, for each query genome any genomes that are too distant to the query genome or too closely related to each other are removed (dotted lines) A). Second, we ensure that at least one genome (bold line) is close enough so that the GIs we identify were inserted from similar time frames and are not biased by the genomes that are currently available B). Also, we require that at least one genome is distant enough from the query genome (bold line) to ensure that the backbone sequences we identify were not inserted recently C). Finally, we require that there be a minimum of 3 reference genomes that have met all other criteria D). The reference genomes that have passed all the cutoffs are used for comparison with the query genome.

Several CVTree distance cutoffs were formulated by us to ensure that appropriate "reference" genomes were selected for comparison to the query genome (Fig. 1A, Fig. 2). These parameters were selected using known groups of strains that are within the proper distance for comparative genomics (e.g. *Pseudomonas aeruginosa *and *Escherishia coli *strains). In addition, the alignments were inspected to ensure that the alignment results were not too sparse or too similar to gain any useful information. A "Maximum Distance Cutoff" of 0.42 was used to remove any genomes that were too distant from the query genome. We observed that at such cutoff, often less than 5% of the genomes can be aligned. A "Minimum Distance Cutoff" of 0.1 was used to remove very closely-related strains that would not provide any additional information and may prevent us from identifying some notable islands that were shared between such closely related strains (Fig. 2A). In addition, by allowing for a larger span of insertion time, this parameter ensures that our method is not limited to identifying only very recently inserted GIs. At least one reference genome must have a distance less than 0.30 from the query genome to ensure that identification of GIs are all within a similar evolutionary time (Fig. 2B). In addition, to ensure that the stable backbone regions identified are ancient enough to be reliable, at least one reference genome must have a distance greater than 0.34 from the query genome (Fig. 2C). Lastly, we require that there be at least 3 suitable reference genomes for each query genome to be used for further analysis (Fig. 2D).

All of these cutoffs can be changed to permit prediction of GIs acquired from different time frames. For example, by increasing the "Minimum Distance Cutoff" and the "Single Close Genome Cutoff" we effectively change the period of time that GI acquisitions are detected, by choosing more divergent genomes for the analysis. Although the inclusion of more ancient GIs could lead to a more comprehensive dataset, it would likely result in an increase in false positives since the proper identification of older evolutionary events can be easily mistaken. However, we did use two additional "relaxed" sets of parameters to determine the effect on GI prediction of changing the default parameters. These relaxed parameters should identify GIs with more ancient origins. The first relaxed set had the same cutoffs as above, except that the "Minimum Distance Cutoff" was changed to 0.15 and the "Single Close Genome Cutoff" changed to 0.34. The second set of parameters was even more relaxed by increasing the "Single Close Genome Cutoff" to 0.20, with all other parameters being the same as the first relaxed set.

Overall, the parameters, in particular the default parameters, were selected to ensure high confidence in the positive and negative datasets, so that they could be used to fairly evaluate the accuracy of several sequence composition based GI prediction tools (see Methods: Comparison of GI tools). The parameters were not changed to maximize the accuracy scores of any GI prediction tools that were evaluated, however default parameters resulted the highest apparent accuracy when GI datasets were compared with a curated, literature-based dataset.

### Detecting GIs using comparative genomics

We used the command line program mauveAligner from the Mauve 1.2.3 software package with its default settings to do all of our whole genome alignments (Fig. 1C) [37]. Mauve allows for genomic insertions, deletions, inversions, and rearrangements and has been used by several researchers for prokaryote genome alignment [38,39]. The query genome was aligned pair wise against each of the reference genomes in the dataset. We used pair wise alignments instead of a single multiple alignment because Mauve 1.2.3 only aligns regions that are present in all genomes. For each pair wise alignment, we extracted regions longer than 8000 nucleotides from the query genome that could not be aligned. Overlapping regions of the query genome that were not aligned in any of the pair wise genome alignments were retained for additional filtering as described below.

One caveat of Mauve is that it enforces a one-to-one alignment so if a duplication event occurs in one of the input genomes only one of the copies will be aligned. We excluded these possible genome duplications and ensured that our putative genomics islands were truly unique to only the query genome, with respect to the reference genomes, by using BLAST similarity search as an additional filter. Each "unique" region was searched against the query genome and all reference genomes using BLAST [40], with default parameters except for an e-value of 1 (instead of 10). All similarity search matches (hits) less than 700 nucleotides were discarded while remaining hits were clustered together if they were less than 200 base pairs a part. Any unique regions that contained clustered hits that covered more than half of the minimum island size (8 kb) were removed and the remaining regions were considered to be putative GIs.

### Detecting highly conserved regions (non-GIs)

In order to develop a negative dataset of regions that do not contain GIs, we identified regions that were conserved across multiple genomes (Fig. 1B). These regions are likely to form the stable backbone of the genomes and are unlikely to be acquired by horizontal gene transfer among the strains considered. A multiple genome alignment of each query genome and all reference genomes previously selected (see Methods: Automated comparative genome picking) was performed using Mauve with minimum backbone length and maximum gap size parameters set to 8000 and 300, respectively. The regions that were conserved across all genomes were extracted from Mauve's backbone output file. Genomic segments that were neither in the positive nor the negative datasets were excluded from further analyses as the origin of these regions are less certain. This helped ensure that whenever we found that a GI predictor made a false GI prediction (i.e. false positive), it was truly very likely false, or whenever it didn't make a prediction, that it was very likely a true GI that it should have predicted (i.e. false negative).

### Comparison of GI tools

Each tool for composition based prediction of GIs was downloaded and if necessary compiled on a computer running a Linux operating system. The tools PAI_IDA [12], AlienHunter [15,29], SIGI-HMM (as part of the Colombo package) [29], Centroid [16], and IslandPath (included both DIMOB and DINUC methods) [8,11], were all run with default values on the query genomes. The precision, recall and overall accuracy of each tool including our own positive and negative datasets was measured using each tool as the "benchmark" dataset. True positives (TP) were counted as any nucleotides that were contained in both the benchmark dataset and the predicted islands. False positives (FP) were those islands that were not predicted by the benchmark (or those overlapping with the negative dataset). False negatives (FN) were counted as all nucleotides within the benchmark dataset that were not in the predicted islands. Precision or specificity was calculated using the standard formula TP/(TP + FP) and recall or sensitivity was calculated using TP/(TP+FN). The equation, (TP + TN)/(TP+TN+FP+FN) was used to measure the overall accuracy.

## Authors' contributions

MGIL designed and implemented the IslandPick pipeline, implemented and analysed the sequence composition based tools, and drafted the manuscript. WWLH implemented some of the sequence composition based tools, collated the literature based GIs, and helped with design of the IslandPick pipeline. FSLB conceived of the study, participated in its design and coordination, and helped to draft the manuscript. All authors read and approved the final manuscript.

## Supplementary Material

Additional file 1 The 118 query genomes and their reference genomes with CVTree distances. Click here for file

Additional file 2 All GIs, with coordinates, predicted using our comparative genomics approach (positive dataset), along with overlap with other GI prediction tools. Click here for file

Additional file 3 Gene lists for each genomic island predicted using IslandPick. Click here for file

Additional file 4 All regions, with coordinates, in the negative dataset, along with overlap with other GI prediction tools. Click here for file

Additional file 5 GIs predicted by each tool and found in the literature, including overlapping regions from other GI tools. Click here for file

Additional file 6 Accuracy measurements for 118 chromosomes listed by each GI prediction tool. Click here for file

Additional file 7 Accuracy measurements for each prediction tool using each tool's GI dataset as the "benchmark". Click here for file

Additional file 8 Average number of GI predictions and accuracy measurements of several GI prediction tools, based on a dataset containing proportionally more ancient islands by using relaxed genome selection criteria. Click here for file

Additional file 9 Accuracy measurements of GI tools for 114 chromosomes, based on a dataset containing proportionally more ancient islands by using relaxed genome selection criteria. Click here for file
