# Supplementary material for: Evaluation of genomic island predictors using a comparative genomics approach
Source: BMC Bioinformatics. 2008 Aug 5;9:329. doi: 10.1186/1471-2105-9-329 (PMC2518932; doi:10.1186/1471-2105-9-329)
Supplement: Additional file 8 — Average number of GI predictions and accuracy measurements of several GI prediction tools, based on a dataset containing proportionally more ancient islands by using relaxed genome selection criteria. [file 1471-2105-9-329-S8.doc]

**Additional File 8** - Average number of GI predictions and accuracy measurements of several GI prediction tools, based on a dataset containing proportionally more ancient islands by using relaxed genome selection criteria. Numbers in brackets represent increase (+) or decrease (-) of the relaxed dataset values compared to the stringent dataset used in the original analysis..

| **Tool** | **Average number of nucleotides in GIs per genome (kb)** | **Precision** | **Recall** | **Overall Accuracy** |
| --- | --- | --- | --- | --- |
| **SIGI-HMM** | 253.8 (+21.1) | 93.3 (+1.0) | 28.1 (-4.9) | 81 (-5.3) |
| **IslandPath/**  **DIMOB** | 186.2 (+15.5) | 83.1 (-2.7) | 25.9 (-9.7) | 79.6 (-6.6) |
| **PAI IDA** | 114.3 (-48.9) | 83.1 (+15.1) | 16.7 (-15.5) | 77.7 (-6.0) |
| **Centroid** | 169.6 (-1.7) | 70.7 (+9.4) | 24 (-3.6) | 77.9 (-4.5) |
| **IslandPath/**  **DINUC** | 457.0 (+12.6) | 57.3 (+2.5) | 41.8 (-11.5) | 77 (-5.2) |
| **Alien**  **Hunter** | 1240.0 (-24.8) | 44.9 (+6.9) | 70.4 (-6.6) | 70.2 (-0.6) |
| **Literature** | 592.6 (+101.3) | 100 (0) | 86.1 (-0.9) | 95.9 (-0.4) |

Results are averaged from 114 chromosomes (see Additional File 9) except for the “Literature” GIs, which were averaged over 4 chromosomes/strains; *Escherichia coli* O157:H7, *E. coli* O157:H7 EDL933, *Salmonella enterica* Typhi str. CT18, and *S. enterica typhimurium* LT2.
